# Implementation research of a cluster randomized trial evaluating the implementation and effectiveness of intermittent preventive treatment for malaria using dihydroartemisinin-piperaquine on reducing malaria burden in school-aged children in Tanzania: methodology, challenges, and mitigation

**DOI:** 10.1186/s12936-022-04428-8

**Published:** 2023-01-06

**Authors:** Geofrey Makenga, Misago D. Seth, Vito Baraka, Bruno P. Mmbando, Daniel P. Challe, Filbert Francis, Athanas Mhina, Daniel T. R. Minja, Mercy Chiduo, Celine Mandara, Edwin Liheluka, Samwel Gesase, Method Segeja, George Mtove, Mathias Kamugisha, Abdallah Lusasi, Frank Chacky, Anna David, Sumaiyya Thawer, Ally Mohamed, Samwel Lazaro, Fabrizio Molteni, Alex Nkayamba, Jean-Pierre Van geertruyden, John P. A. Lusingu

**Affiliations:** 1 National Institute for Medical Research, Tanga Centre, Tanga, Tanzania; 2 National Malaria Control Programme, Dodoma, Tanzania; 3 Swiss Tropical and Public Health Institute, Allschwill, Switzerland; 4 University of Basel, Basel, Switzerland; 5 Tanzania Medicines and Medical Devices Authority, Dar es Salaam, Tanzania; 6 Global Health Institute, University of Antwerp, Antwerp, Belgium; 7 Centre for Medical Parasitology, Institute of Medical Microbiology and Immunology, University of Copenhagen, Copenhagen, Denmark

**Keywords:** Malaria, Effectiveness, Dihydroartemisinin-piperaquine, Implementation research, Anaemia, Cluster randomized trial, Evaluation, Operation challenges, Mitigation

## Abstract

**Background:**

It has been more than 20 years since the malaria epidemiologic shift to school-aged children was noted. In the meantime, school-aged children (5–15 years) have become increasingly more vulnerable with asymptomatic malaria prevalence reaching up to 70%, making them reservoirs for subsequent transmission of malaria in the endemic communities. Intermittent Preventive Treatment of malaria in schoolchildren (IPTsc) has proven to be an effective tool to shrink this reservoir. As of 3^rd^ June 2022, the World Health Organization recommends IPTsc in moderate and high endemic areas. Even so, for decision-makers, the adoption of scientific research recommendations has been stifled by real-world implementation challenges. This study presents methodology, challenges faced, and mitigations used in the evaluation of the implementation of IPTsc using dihydroartemisinin-piperaquine (DP) in three councils (Handeni District Council (DC), Handeni Town Council (TC) and Kilindi DC) of Tanga Region, Tanzania so as to understand the operational feasibility and effectiveness of IPTsc on malaria parasitaemia and clinical malaria incidence.

**Methods:**

The study deployed an effectiveness-implementation hybrid design to assess feasibility and effectiveness of IPTsc using DP, the interventional drug, against standard of care (control). Wards in the three study councils were the randomization unit (clusters). Each ward was randomized to implement IPTsc or not (control). In all wards in the IPTsc arm, DP was given to schoolchildren three times a year in four-month intervals. In each council, 24 randomly selected wards (12 per study arm, one school per ward) were chosen as representatives for intervention impact evaluation. Mixed design methods were used to assess the feasibility and acceptability of implementing IPTsc as part of a more comprehensive health package for schoolchildren. The study reimagined an existing school health programme for Neglected Tropical Diseases (NTD) control include IPTsc implementation.

**Results:**

The study shows IPTsc can feasibly be implemented by integrating it into existing school health and education systems, paving the way for sustainable programme adoption in a cost-effective manner.

**Conclusions:**

Through this article other interested countries may realise a feasible plan for IPTsc implementation. Mitigation to any challenge can be customized based on local circumstances without jeopardising the gains expected from an IPTsc programme.

*Trial registration* clinicaltrials.gov, NCT04245033. Registered 28 January 2020, https://clinicaltrials.gov/ct2/show/NCT04245033

**Supplementary Information:**

The online version contains supplementary material available at 10.1186/s12936-022-04428-8.

## Background

Since 2000, the malaria burden in sub-Saharan Africa has declined due to intensification and scaling up of control interventions [1]. However, this decline has catalysed a shift in the malaria burden toward children above 5 years [2–5]. In high-transmission settings, up to 70% of school-aged children harbour malaria parasites [6]. Most of these children are asymptomatic, though malaria accounts for somewhere between 13 and 50% of all school absenteeism [7]. Thus, from an epidemiological point of view, school-aged children contribute substantially as parasite reservoirs to onward malaria transmission in the population [8].

According to the National Malaria Control Programme (NMCP), malaria prevalence in Tanzania declined from an average of 18.1% in 2008 to 7.0% in 2017, marking an epidemiological transition from meso-endemic to hypo-endemic levels. However, this decline varies across and within regions and/or councils [9]. The nationwide school malaria survey conducted between 2014 and 2015 reported an overall prevalence of malaria of 21.6% by malaria rapid diagnostic test (mRDT), and ranged from < 0.1% to 53.0% among regions and from 0% to 76.4% among councils [3]. Therefore, the NMCP developed a Supplementary Malaria Midterm Strategic Plan (SMMSP 2018–2020) to customize malaria interventions by stratifying the burden of malaria in Tanzania mainland [9] in order to achieve goals set in the 2020 strategic plan and malaria elimination by 2030 [3, 10]. The SMMSP endorsed the implementation of the Intermittent Preventive Treatment in schoolchildren (IPTsc) in high endemic areas where, it predicted IPTsc to have limited additional benefit on lowering prevalence but with higher impact on malaria cases averted. The SMMSP recommended the use of artemisinin-based combination therapy (ACT) alternative to the first-line treatment, and suggested dihydroartemisinin-piperaquine (DP) for such implementation.

The presence of a national school health programme (NSHP) that combines the schistosomiasis and soil transmitted helminths (STH) control packages under an integrated neglected tropical diseases (NTD) programme [11], sets a precedent for future integration of malaria interventions into school health programmes [11, 12]. In addition, due to high primary school enrolment rate, schools act as platforms for health intervention delivery, including anti-malarial efforts. This study aim to explain the methods, challenges and mitigations used to evaluate the implementation of IPTsc using DP given three times a year, which provided evidence on the operational feasibility and impact of IPTsc on clinical malaria incidence in a high endemic area in Tanzania. The study also determined acceptability, impact of IPTsc on haemoglobin levels and asymptomatic parasitaemia in school-aged children. Similar methods can be adopted and replicated by other countries intending to implement IPTsc as per current World Health Organization (WHO) recommendation [13].

## Methods

### Study area

**Fig. 1:**
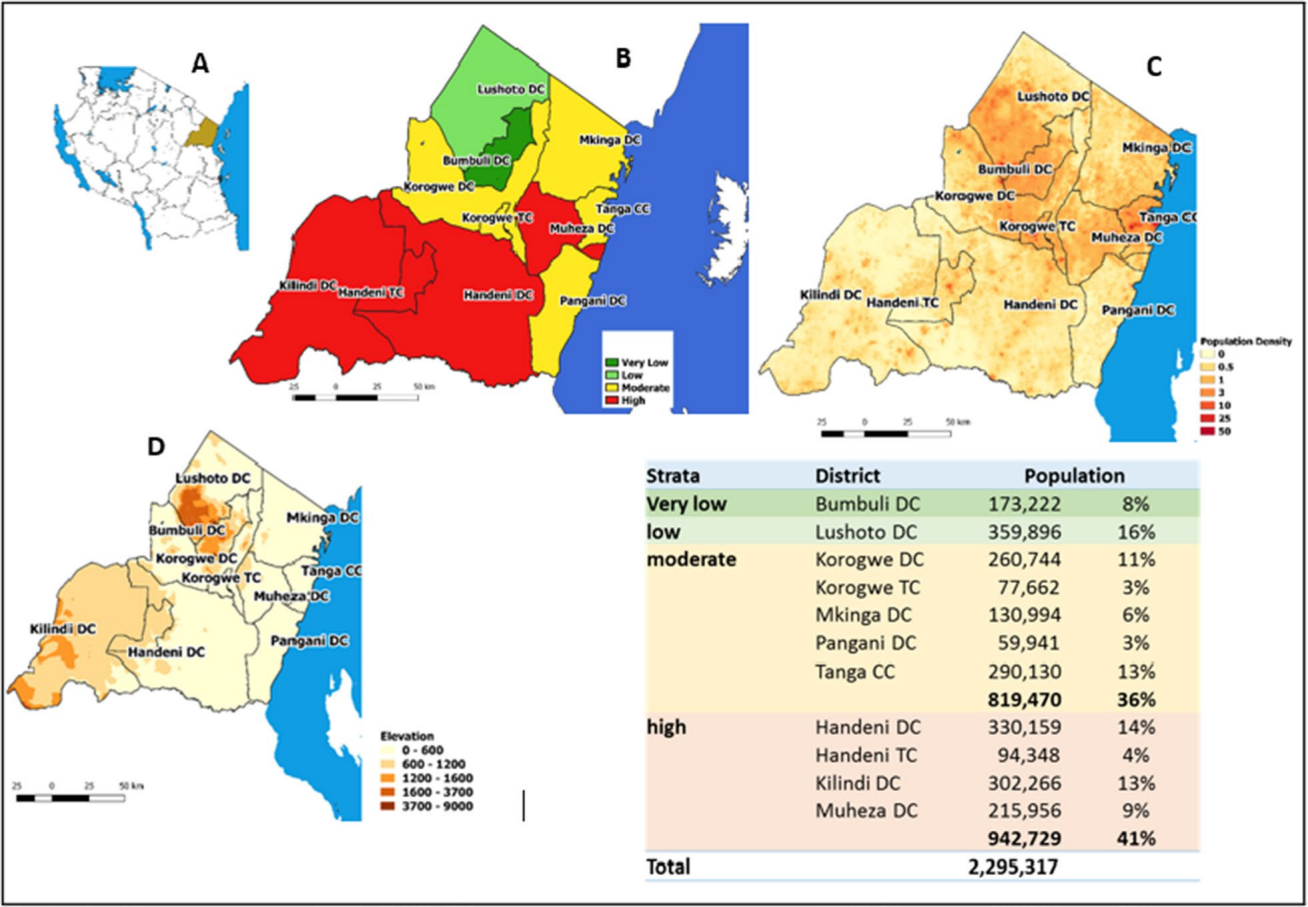
Map of Tanzania (**A**) and Tanga Region showing malaria endemicity (**B**) Population density (**C**) and elevation (**D**). The table shows population distribution in each council of Tanga Region categorized per malaria endemicity

**Fig. 2:**
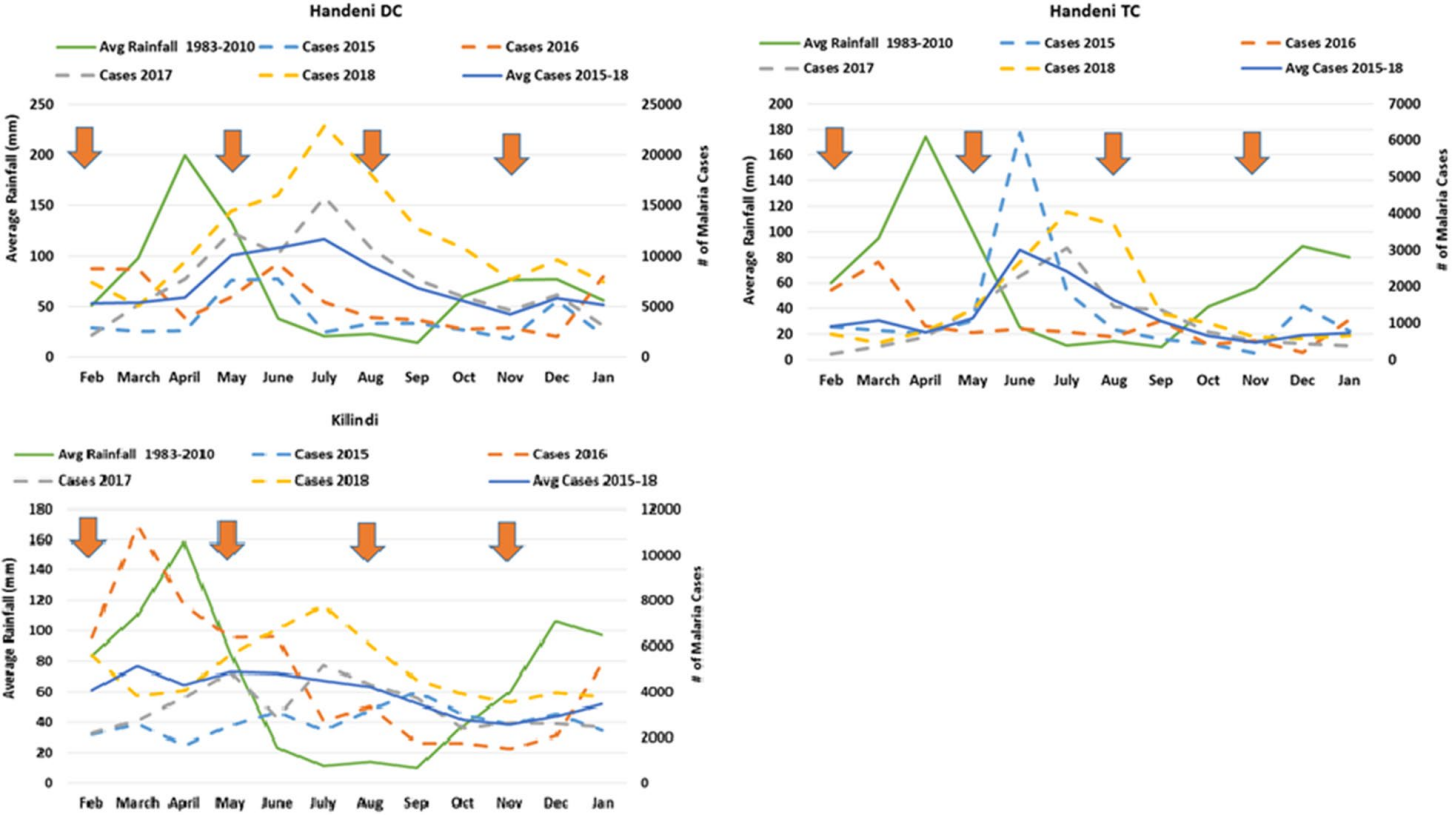
Malaria cases and rainfall trends per council

The project was conducted in Handeni District Council, Handeni Town Council (Handeni DC and TC) and Kilindi District Council (Kilindi DC) of Tanga Region, northeastern Tanzania. The three councils border each other and have been classified as high malaria endemic strata by the NMCP [9] (Fig. 1). Malaria transmission in this site occurs year-round, with two seasonal peaks following the long rainy season from March to May and the short rainy season from November to December (Fig. 2). However, there are variations in malaria incidence among the three councils. In 2018, NMCP data showed Handeni DC had a much higher malaria incidence (413/1000 population) than the other sites (Handeni TC- [183/1000] and Kilindi DC [187/1000]). The existing mainstay for malaria control includes use of long-lasting insecticide-treated nets (LLINs) and prompt diagnosis and treatment with ACT, mainly artemether-lumefantrine (AL) as the first-line treatment for uncomplicated falciparum malaria [14]. The intermittent preventive treatment of malaria in pregnancy using sulfadoxine-pyrimethamine (IPTp-SP) has also been implemented as a policy countrywide per WHO recommendation [14, 15].

As of 2018, Handeni DC, Kilindi DC and Handeni TC had 117, 111, and 33 public primary schools that enrolled 69,454, 61,258, and 19,549 schoolchildren, respectively. The three councils had 52 wards (20 in Handeni DC, 20 in Kilindi DC, and 12 in Handeni TC). There were only five private primary schools across the three councils, which enrolled a total of 627 schoolchildren. The study enrolled public primary schoolchildren from selected wards in the three councils with health teachers who had been involved in several mass drug campaigns implemented at schools by the NTD programme.

### Study design

This was an effectiveness-implementation hybrid trial [16–19] to assess feasibility and effectiveness of IPTsc using DP against standard of care (control). Wards in the three study councils (Handeni DC, Handeni TC and Kilindi DC) were the randomization units (clusters). Each ward was randomized to implement IPTsc or not (control). In all wards in the IPTsc arm (equivalent to half the number of wards in the councils), the interventional drug (DP) was given at an interval of four months, three times a year (March, July and November) in liaison with school schedules and seasonal transmission peaks (Fig. 2).

**Fig. 3:**
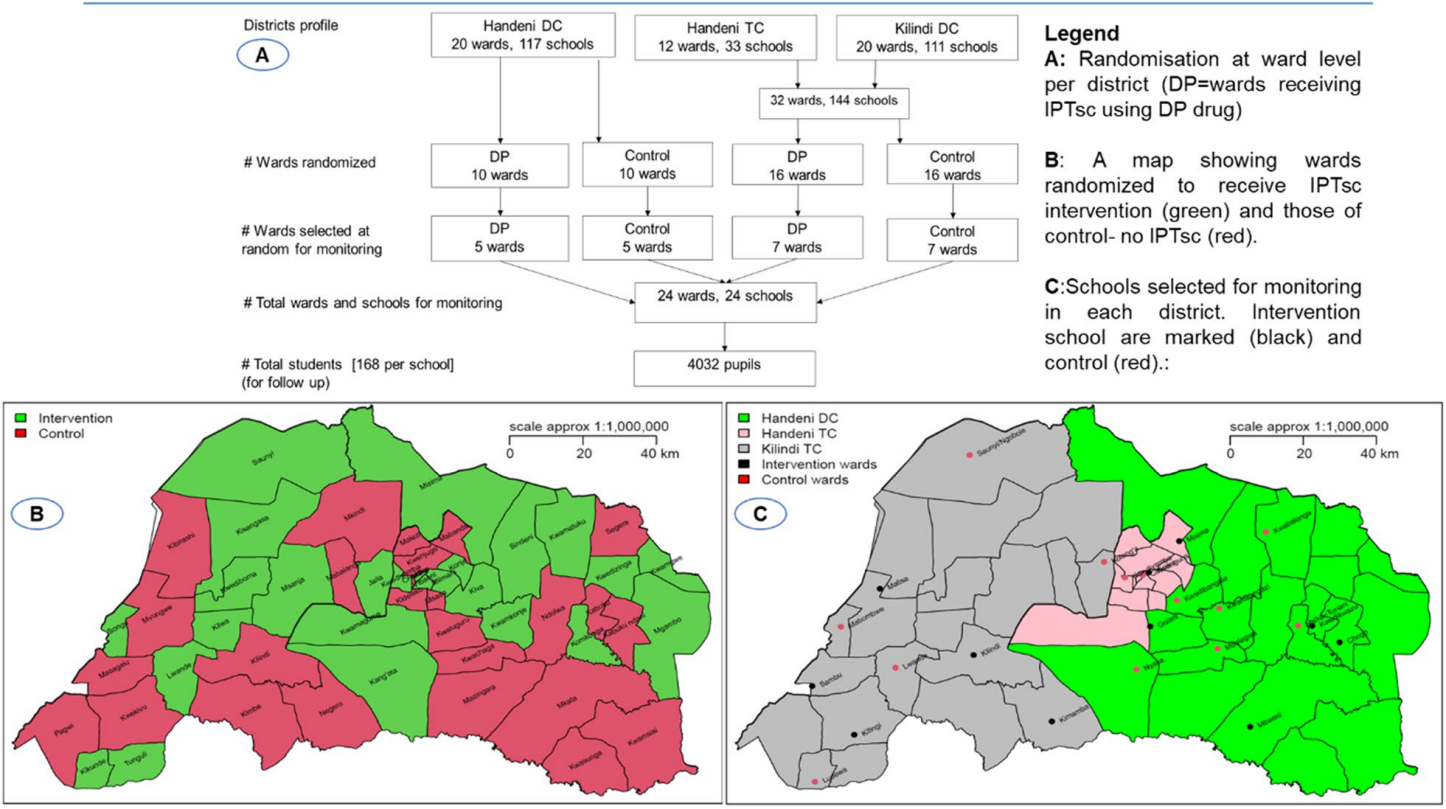
Study design and ward allocation for intervention in three councils of Handeni DC, Handeni TC and Kilindi DC, Tanga, Tanzania

For study evaluation on the intervention effectiveness, wards were selected from each council at random and distributed equally per study arm to act as representatives in the evaluation (Fig. 3A). In these representative wards, one school was selected at random to provide evaluable children in the respective intervention. Following random selection, each school was anticipated to contribute 168 schoolchildren (a total of 4032 across all councils) to participate in monitoring or evaluation of the intervention under close supervision. Participants who became sick from uncomplicated malaria were treated with AL according to national treatment guidelines [14, 20]. Finger prick blood samples were collected from randomly selected schoolchildren for malaria microscopy (at baseline and during the last visit), and haemoglobin concentration measurement (at each visit) to determine prevalence of malaria and anaemia, respectively. Monthly reports on absenteeism and reported adverse events were collected in collaboration with schoolteachers, local health facility workers and the local community health workers (CHW).

### Description of the intervention (study drugs)

IPTsc aims to treat and prevent malaria infections in school-aged children. The practice requires the administration of a full treatment course of an anti-malarial medicine at regular intervals to these children [13]. This will clear both asymptomatic and symptomatic malaria infections and, in turn, avoid the consequences of infection (e.g. anaemia, absenteeism, cognitive impairment) in school-aged children who are at high malarial risk [21–23]. Evidence on the effectiveness and feasibility of the IPTsc strategy is robust in most sub-Saharan African countries [24]. As of June 3rd, 2022, the WHO recommends the rollout of IPTsc in moderate to high endemic transmission settings with *Plasmodium falciparum* parasite prevalence greater than 10% or an annual parasite incidence greater than 250 per 1000 [13]. However, these thresholds are not regarded as absolutes for determining applicability of the IPTsc recommendation [13].

A fixed-dose combination (FDC) of DP (D-Artepp by Guillin Pharmaceuticals, China) has been approved by the European Medicines Agency (EMA). DP is registered by the Tanzania Medicines and Medical Devices Authority (TMDA) as an alternative drug for treatment of uncomplicated malaria. This drug is safe and efficacious at a body weight-associated dose of 60–73.9 mg/kg, given once daily over 3 days for uncomplicated malaria [25, 26]. DP’s partner drug, Piperaquine (PQ) has a long elimination half-life [27].

### Intervention assignment and selection of schools for close monitoring

During the preparatory visit, the study team met with local Council Health Management Team (CHMT) members in each council. In these meetings, randomization was conducted following a brief yet comprehensive explanation of the planned study. This explanation was critical to ensure the whole randomization process transparent to the local government officials. The list of wards in each council was entered into an Excel sheet from which a randomization code was made. The first half of selected wards were assigned to the IPTsc arm, while the remaining were assigned to the control arm. Again, in each study arm, half of the wards were randomly selected for effectiveness assessment; only one school was selected after randomization of the schools in the respective ward. Here, randomization was done by mixing small folded papers with one school’s name on each piece. One of the CHMT members was then given an opportunity to pick any one of the mixed papers and read aloud the name of the school written on it. At this level, schools that were located in hard-to-reach areas were excluded from randomization following advice from the local authorities regarding convenience and logistics.

## Implementation procedures

### Community and local government engagement approach

**Fig. 4:**
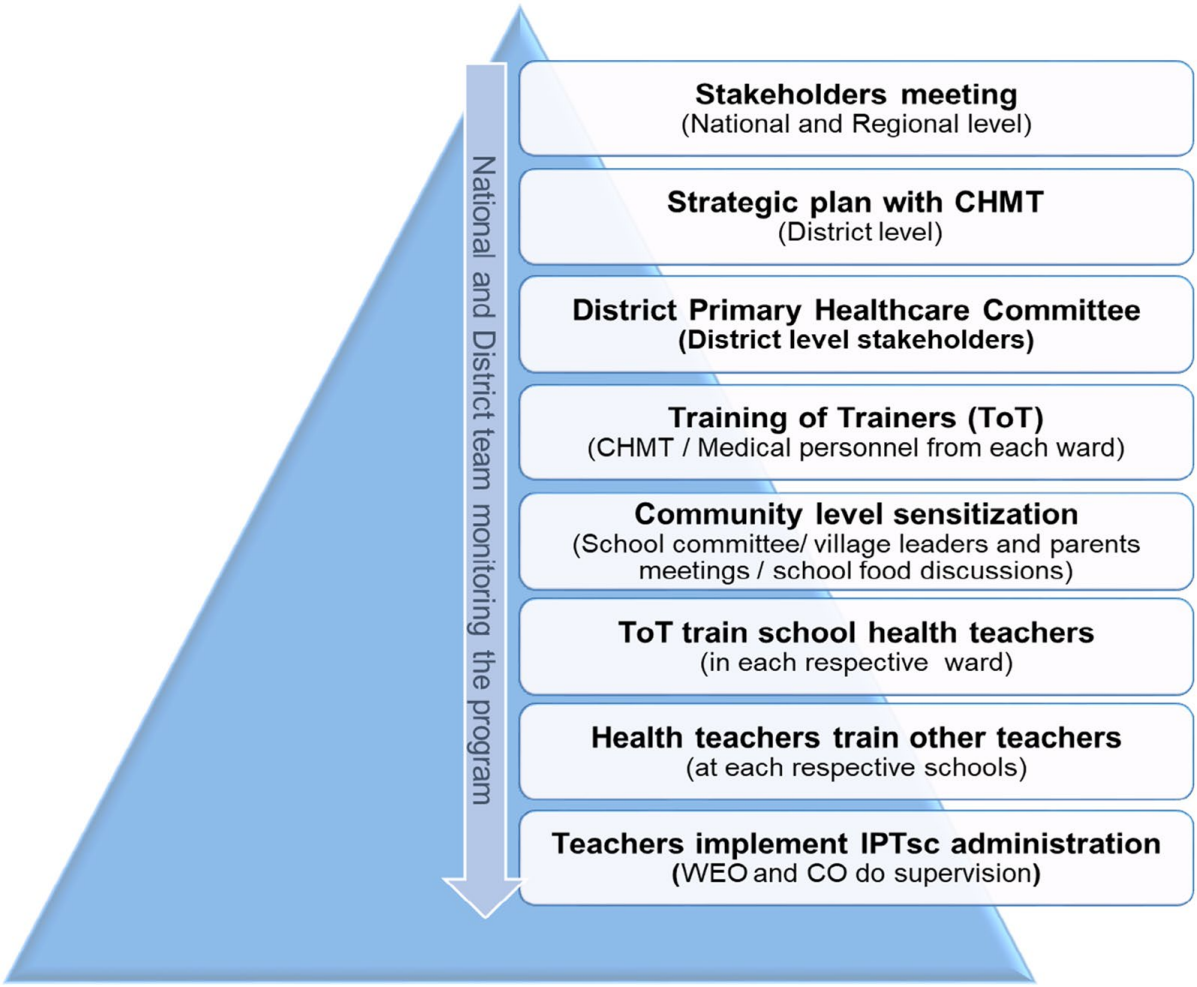
Strategic approach for community engagement to IPTsc implementation. Legend; (CHMT = Council Health Management Team, WEO = Ward Education Officer, CO = Clinical officer)

The study implementation was evaluated alongside possible integration with other existing school health intervention programmes [28]. From the very beginning of study setup, the CHMT in each council was involved in a manner similar to that described by the NTD deworming programme. Along with the District Malaria Focal Person (DMFP), the District NTD coordinator, District Pharmacist, and the District school health coordinator (DSHCo), the study team developed a strategic plan for implementing IPTsc in each council. The resultant plan was to approach the communities via the district primary health care committee (DPHC) chaired by the District Commissioner in order to encourage local leaders to promote community awareness of IPTsc in various arenas including churches, mosques, school committees and parents’ meetings, while also gaining local permission for intervention. At the school/village level, the study team invited village government leaders to centrally located sensitization meetings with school committees. The study team made regular visits at selected wards and schools, where collaborations with the Ministry of Health (MoH), the Ministry of Education, Science, Technology and Vocational Training (MESTVT) and the President's Office Regional Administration and Local Government (PO-RALG) at national and local levels were established. Figure 4 summarizes how this approach was made.

### District Primary Healthcare Committee (DPHC) meetings

Two DPHC meetings were conducted, one in Handeni (TC and DC), and the other in Kilindi DC. Chaired by the respective District Commissioners, the DPHC is mandated to safeguard health interests in the district. Members include all district council government leaders, together with heads of departments, key opinion personnel and religious leaders, department heads and, key opinion personnel and religious leaders. During these meetings, the IPTsc protocol was presented, discussed and approved for implementation in the three respective councils. DPHC members were urged to collaborate with the team in sensitising the communities under their authority.

### Training for IPTsc implementation

The study team conducted Training of Trainers (ToT) seminar, where a team of District/Council health workers were trained on how to facilitate training of both primary school health teachers as well as Head teachers on the procedures for DP drug administration, accountability and adverse drug reaction (ADR) monitoring in schoolchildren. The Pharmacovigilance department of the TMDA provided training on adverse drug reaction [29] reporting using the well-known yellow ADR reporting forms which can be posted free of charge to TMDA headquarters through the Tanzania Posts Corporation (TPC). This was done to ensure that teachers and CHWs are able to utilize the available tools for pharmacovigilance after the study becomes a policy. After training, two trainers were chosen for each ward who then gathered three teachers per school (2 school health teachers and 1 head teacher), and met at ward offices or at a centrally located school. The trained teachers then supervised and trained their fellow teachers at their respective schools. The health facility in-charge in collaboration with the Ward Education Officer (WEO) supervised these trainings under guidance from (National Institute for Medical Research) NIMR and CHMT (malaria team).

### Medicines accountability procedures

Drugs were distributed by MSD at the district council level, where the District Pharmacist then managed distribution to local health facilities near primary schools. The pharmacist recorded drugs received from MSD and drugs given out to local health facilities. The clinical officer in-charge at the local health facilities accounted for drugs dispensed to schools (doses estimated per number of pupils in a school) by filling a special drug accountability form (Additional file 1: Appendix S1). School health teachers filled out their respective drug-dispensing logs (Additional file 2: Appendix S2) at school level. The drug-dispensing logs were created similarly to those used in NTD programmes. Appropriate standard operating procedures (SOPs) were adhered to by the pharmacist, health facility in-charge and the schoolteachers dispensing drugs to schoolchildren. The drug accountability form and the drug-dispensing logs were used for drug reconciliation in accountability of all drugs received, dispensed or destroyed in case of expiration or damage. All study drugs either received in from MSD or sent to schools were verified by the investigator or designee; the amount sent and the exact supplies received were documented by signing and dating the appropriate shipping documents. Shipments to schools considered batch numbers to ensure no school obtained more than one batch in a single administration round.

### Criteria for receiving IPTsc drugs

The implementation mimicked a real-life scenario, where all children (regardless of sex), aged 5 years and above in class 1 to 7 in public primary schools of selected wards were eligible for IPTsc. However, for safety concerns, drugs were not given to children known to be allergic to ACT, on treatment for chronic heart disease, or those who were taking anti-malarial drug in a week coinciding with IPTsc administration or who had just finished an anti-malarial course within a week prior to IPTsc administration (Additional file 3: Appendix S3).

### Treatment administration and assessment of compliance

During the week prior to study drug administration, all schools received log forms to enable teachers to register their schoolchildren and further sensitize pupils and parents. During sensitization, which was normally conducted through parental meetings at schools, it was emphasized that parents whose children are currently taking anti-malarial treatment or who have just finished within a week’s time, should notify the teachers before the announced date for drug administration at schools. During registration, children were weighed on scales provided by the study team. Thus, schoolchildren received drugs according to their respective body weight, following the manufacturer’s manual. Drugs were taken with water, one to two hours before lunch was served. Children were observed for 30 min post-drug administration. If a child vomited within this time, he/she was given another dose and the information was documented. If a child vomited the study drug a second time, he or she was withdrawn from receiving the drug.

In the event a child missed IPTsc medication on day one, they were given an opportunity to start on the second day of the IPTsc round. If a pupil missed the second day’s dose, the child was followed home by the CHW for information and administration of the second dose (this could be within the same day or on the third day). If a child missed two consecutive doses (i.e. day 2 and 3) then this was considered an incomplete course.

In this study, at least seven teachers were deployed depending on the number of pupils in a school. Each class teacher administered drugs to his/her class for all doses. This shortened delivery time to ensure routine school activities were not disrupted by the IPTsc programme, thereby increasing adherence. This arrangement also guaranteed that delivery of all IPTsc study drugs was directly observed for maximum compliance assessment (Additional file 3: Appendix S3).

### Safety assessment

The TMDA defines an ADR, as an episode which is noxious and unintended, and which occurs at doses normally used in humans for the prophylaxis, diagnosis or treatment of disease or for the modification of physiological functions [30]. In this study, all affected children (directly or through their parents/guardians) were encouraged to report ADRs directly to their school health teachers, CHWs or healthcare workers at nearby facilities. Teachers, CHWs and health care workers filled out the TMDA ADR reporting forms (ARRT Additional file 4: Appendix S4) and sent them to TMDA as directed on the form. This process was expected to create more awareness on pharmacovigilance in the communities involved. Teachers kept records of all ADRs reported to them and forwarded this information to TMDA. Thereafter, the DMFP accessed the collected information for reporting to NMCP and NIMR. Teachers were encouraged to contact or direct parents/guardians to the local health facility for free management of any reported ADR during the IPTsc administration week.

### Study supervision and quality assurance

During supervision, the team comprised officials from NIMR, NMCP, TMDA (pharmacovigilance) and some members of CHMT designated as the malaria team (consisting of the DMFP, DSHCo and the DNTDCo). Supervision was done by both randomly selecting wards and encompassed schools, and targeted supervisory visits following a reported adverse event or any irregularity in IPTsc conduct, e.g. a delay in drug dispensing. A special supervision form was used to document the delivery process, including how teachers fill out the dispensing logs, drug handling, and any matter arising such as reporting ADR (Additional file 4: Appendix S4). If communication were to be made, supervisors would use both phone calls and social media working groups created for IPTsc programme. This helped convey the message promptly to all members.

### Local supervision and collection of coverage information

In all wards with IPTsc intervention, the WEO and respective health facilities’ clinical officers in charge were responsible for supervising the IPTsc implementation in their respective catchment areas. They ensured vital materials such as drugs and forms were delivered to each school, and that meetings with school parents and guardians were organized for continuous community sensitization, including explanation of eligibility for taking drugs as stipulated on drug dispensing information sheet (Additional file 3: Appendix S3).

During drug dispensing in each IPTsc round, class teachers, head teachers and WEO filled a special coverage assessment form (Additional file 5: Appendix S5). The information gathered included the following: number of children in a class/school/ward, number of children who completed the three-day course (therapeutic full dose) of DP, number of children with incomplete dosing, and the number of missing children with reasons for noncompliance such as sickness, drop out, ongoing anti-malarial treatment and/or refusal. A class teacher then provided a summary for his/her class, which the head teacher then combined with other class summaries for a complete school report. The WEO continued this chain by combining all school reports to create a ward report. Finally, the study team collected all forms filed by the class teacher, head teacher and the WEO to compile a council report.

## Evaluation of IPTsc effectiveness

### Sample size calculation

For the intervention evaluation, malaria incidence in school age children from Handeni was assumed similar to that derived from the Demographic Health Information System 2 (DHIS2) for the same district council (413 cases per 1000) and that the incidence would be reduced by 40% following intervention when compared to control. A sample size of 140 schoolchildren was to be followed from each cluster/school to document cases of malaria. The study assumed correlation coefficient k = 0.2, a power of 80% and type one error (α) of 0.05. Hence, five clusters were required for each of the intervention and control arms. Similar parameters were assumed for Kilindi DC and Handeni TC, but with slightly lower incidence (183 cases per 1000). In this, seven clusters/schools (distributed basing on number of wards ratio per council; 5 from Kilindi DC and 2 from Handeni TC) were required for each of the intervention and control wards from the two councils. To adjust for loss to follow-up, an attrition rate of 20% was considered from each ward/school. Therefore, a total of 4032 schoolchildren from 24 schools (i.e.1680 from Handeni DC, 672 from Handeni TC and 1680 from Kilindi DC) were expected to be enrolled in the study (Fig. 3A).

### Enrolment and follow up procedure

At schools selected for close monitoring, participants for effectiveness evaluation were selected at random among schoolchildren of class 1 to 6. Each class contributed 30 schoolchildren balanced by sex. Here, systematic random sampling was conducted, wherein boys and girls organized separately and a respective total number of schoolchildren (i.e. boys or girls) was divided by 15 to obtain the counting interval. A starting number was chosen from a random Excel-generated list of counting numbers (1 to 9) that was created for each school. Participants were then selected starting with an identified random number then followed by counting the interval obtained in between each new selection until the necessary number of participants, 15 per sex, had been achieved. In the event that a class had an even number of girls or boys or had less than 30 schoolchildren total, then another class with a greater number of schoolchildren would compensate for the missing ones (Additional file 6: Appendix S6).

Additionally, for schoolchildren selected for evaluation of effectiveness of IPTsc, an informed written consent was obtained from their parent or guardian, which included an assent if a child was 11 years or older. For this evaluation, schoolchildren aged 5 to 15 years and of class one to six were eligible for enrolment. Parents, guardians and schoolchildren were informed of their right to withdraw from the study at any time. Baseline data on social demographics and well-being of schoolchildren were obtained from parents or guardians who had signed consent. At this stage, home visits were necessary for acquisition of global positioning system (GPS) satellite location data and verification of social demographic data with regard to household characteristics. Given that only a portion of schoolchildren were to be enrolled for effectiveness evaluation, the team made one school visit a day before the school parents meeting, where possible eligible children were selected at random (at least 30 children per class, extras were chosen in case a parent could not attend the meeting), and told to bring their parents to a school meeting.

**Fig. 5:**
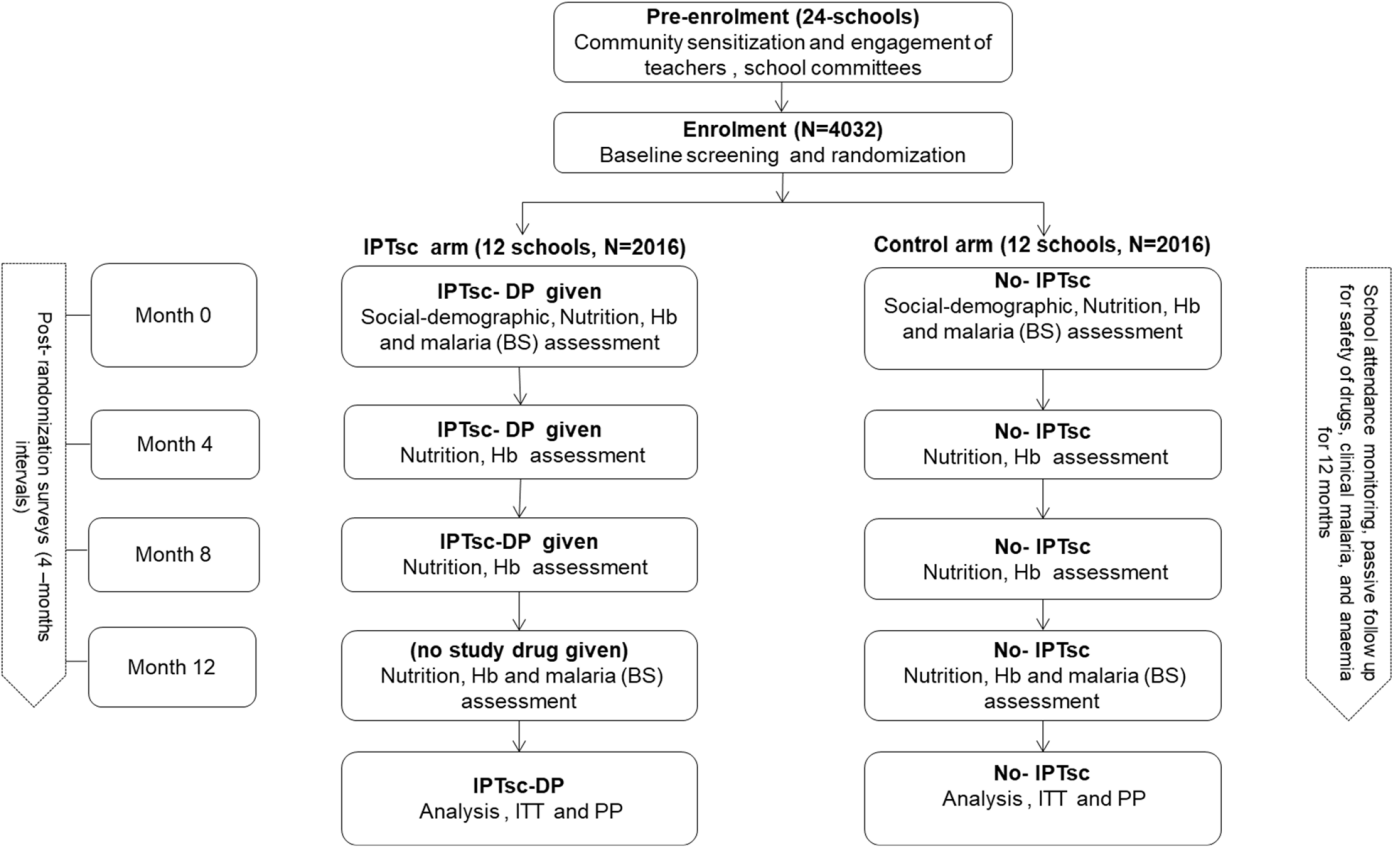
IPTsc effectiveness assessment flow chart. Legend: IPTsc = intermittent preventive treatment in school-aged children; DP = dihydroartemisinin-piperaquine; Hb = Haemoglobin concentration in g/dl; BS = Blood slide; ITT = intention-to-treat analysis; PP = per-protocol analysis

During the parents’ meeting, the study was explained in detail before a written informed consent was administered to those interested. If the parent or guardian of a pre-selected child missed a briefing meeting, he/she was followed up at home for briefing and consenting processes. Nonetheless, parents or guardians who had shown interest during the meeting and whose children were in the pre-defined list, were given priority to replace those who were either absent or did not show interest in the study. This was done to attain the required number of children per school, balanced by sex and class year, from whom biological benchmarks (i.e. reduction of parasitaemia and anaemia prevalence) were to be evaluated through routine testing for malaria and haemoglobin levels at four-month (a total of four visits) (Fig. 5).

### School attendance and clinical malaria monitoring

During scheduled study visits, all children with symptoms (fever) were screened for malaria, regardless of study arm. If a participant missed school on the day of follow-up visit, the study team would conduct a home visit for the participant. In addition, two CHWs were assigned to each school selected for evaluation of IPTsc effectiveness; these were trained to diagnose and treat uncomplicated malaria using the standard guidelines. During follow up, if an enrolled child was absent, school health teachers were encouraged to contact the CHWs in their respective villages, who would then pay a home visit seeking reasons for the absenteeism. The information would be logged on a special attendance monitoring form (Additional file 7: Appendix S7) by school health teachers. Any sick child, together with his/her parent or guardian, was advised to consult the CHW for a preliminary assessment and documentation of the events. In this assessment, the CHW would test for malaria using RDT. If malaria was diagnosed, the CHW would issue treatment according to the national malaria treatment guidelines [14]. All RDT-negative patients and RDT-positive children with severe symptoms were referred to a nearby health facility for further treatment. This was done to not only accelerate diagnosis and treatment of malaria, but also ensure proper recording of all of participant’s malarial episodes. This service was free of charge to participants.

To avoid duplicate information, all referred cases were given a special referral form (Additional file 8: Appendix S8) that was recognized at the health facility. Given the information on the referral form, the health facility would not repeat rapid testing. Avoidance of redundant testing and diagnosis were especially important because the CHWs were supplied with RDTs and anti-malarial drugs (AL) from the same health facilities to which they referred complicated cases. The CHW would present a list of participants who were tested for malaria every week to a clinical officer in charge. The clinicians would then verify the test results and issue another pack to replace those already used. Health workers at the facilities in the study area were responsible for documenting the cases of malaria that were experienced by sick participants who happen to have attended at respective health facilities. Case record forms (Additional file 9: Appendix S9) with incentives were presented to health workers for this purpose.

### Laboratory methods

Thick and thin blood smears were obtained both prior to treatment at baseline and month 12 from all participants of the IPTsc effectiveness evaluation scheme. Malaria parasitaemia was confirmed after double examination by expert microscopists to verify the presence of *Plasmodium* species and calculate the parasite density. Each expert was blind to the readings and conclusions of another. Haemoglobin concentration was measured at recruitment and during scheduled follow up visits using a haemoglobinometer (HemoCue AB, Sweden). Finger-prick blood (dried blood spot-DBS) samples were collected at every visit and prepared on Whatman 3 M filter paper, air-dried and stored in plastic bags containing desiccant and preserved in a -20ºC freezer. All samples were processed and then archived in the Amani Biomedical Research Laboratory (AMBRELA) at NIMR Tanga Centre. Samples were labelled without revealing the randomization group of a participant. The DBS samples will be used for the detection of sub microscopic parasitaemia (by polymerase chain reaction-PCR), detection of markers of drug resistance, as well as future host-parasite genetic studies to examine malaria transmission trends and the impact of IPTsc.

### Qualitative research: evaluation of acceptability

To determine acceptability of the study strategy, including community and frontline caregivers’ perceptions of the recommended drug combination usage, qualitative research was conducted following the final round of IPTsc administration. Questionnaire and interview topics were adapted to the respondent type and included: socio-demographic details, experiences with IPTsc, perceptions of IPT, ideas about malaria and malaria prevention, and potential bottlenecks in implementation of the IPT strategy following the RE-AIM framework as described elsewhere [31–35]. Respondents included study participants/parents, teachers, district officials (DNTDCo, MFP, DSHCo), and CHWs. Four focus group discussions (FGDs) per school were conducted for those who received the intervention; two for schoolchildren (one for boys and one for girls) and parents/guardians (one for females and one for males). Between 5 and 10 randomly selected schoolchildren and 5–10 parents or guardians were involved in study evaluation at each school comprising a mixture of those who participated in close follow up for IPTsc effectiveness evaluation and those who did not. In addition, in-depth interviews (IDIs) were conducted in each school with two school health teachers and one CHW. Moreover, IDIs were conducted purposefully with district council officials responsible for malaria, NTD, and school health programmes. Data collection tools were pre-tested with a number of respondents by the investigators and research assistants to ensure comprehension and appropriateness. With the consent of participants, IDIs and FGDs were audio-recorded and transcribed verbatim. The investigator and research assistant also kept field notes on their observations of matters arising during the interviews.

### Concomitant treatments

During enrolment and follow up, paracetamol (acetaminophen) was administered to all participants with fever (> 37.5 °C). This, together with any other medication provided for treatment during visits or taken by the participant during the study period, was recorded on the appropriate section of the Case Report Form (CRF) and/or adverse event (AE) form.

### Ethical considerations

The study obtained ethical approval from the Medical Research Coordinating Committee (MRCC, Tanzania) with approval number NIMR/HQ/R.8a/Vol.IX/3291 and NIMR/HQ/R.8c/Vol.I/1652 (for extension) and regulatory approval from the Tanzania Medicines and Medical Devices Authority (TMDA) with approval number TMDA0019/CTR/0018/03. Informed consent was obtained from parents /guardian of schoolchildren involved in effectiveness evaluation. The study is registered on clinicaltrials.gov with registration number NCT04245033.

## Results

### Data management

Data from the implementation research mainly relating to IPTsc coverage and adverse drug reactions were collected through special paper forms (drug accountability and dispensing forms). These forms were crosschecked prior to their dispatch to wards and council level for compilation. The head teacher signed and stamped their final forms before sending them to WEO, who also checked before completing compilation of a ward report. All these forms were then sent to the council, where the study team entered the information into the Open Data Kit (ODK) toolkit. ADR reporting forms were directly sent to TMDA (or collected at site by TMDA team) and then entered into the TMDA pharmacovigilance platform via an account created for this study in the TMDA-WHO Vigflow for Pharmacovigilance (https://vigiflow.who-umc.org/).

For the effectiveness evaluation portion, data from baseline household surveys, routine clinical evaluation visits and adverse events were entered directly into ODK applications installed on tablets. Spatial data (point coordinates for schools and households) were collected during household and school visits using the GPS capabilities of ODK. To ensure data accuracy of data, all sections (demographic, clinical and laboratory) were completed by trained and qualified personnel. Electronic forms were coded, and filters and skip patterns were set to minimize chances of errors. The filters were used to restrict entry of unwanted or incorrect information into the database. At the central point, data managers ran queries to check the correctness of data entered. Where errors flagged, queries were sent to the field manager for appropriate actions. Paper-based data (from laboratory results) were verified and double entered into a database developed in Microsoft Access 2010 (Redmond, WA, USA), while consistence checks and analyses were done using STATA software version 15.0 (StataCorp LP, TX, USA). Quality control and data assurance of the data were maintained at all stages following site standard operating procedures (SOPs).

All responsible personnel had unique codes for data entry, and access to the database was extended as a privilege by the system administrator. Data managers had broad database access while others had restricted access depending on roles assigned. For instance, data entrants could enter new data but not change extant entries, while field managers had the ability to respond to queries raised by the data manager. Any changes done to the database were documented.

Back-ups of data were done on a daily basis onto external hard disks that were stored in a secured place separate from the building hosting data management section. A training programme and procedures were implemented before the data systems were fully deployed. Ongoing management including re-training of the system was also observed throughout the lifetime of the project. An enormous amount of data has been generated from this study, and these will benefit different stakeholders in the future, including university students. Such students will be invited to use the data in line with policies on data sharing, including the Data Transfer Agreement (DTA) [36] which facilitates sharing of data for the purpose of informing the public of intervention effect as well as advancing the knowledge body with new analysis.

### Statistical analysis of study endpoints

#### Implementation research

Tabulations and plots were used to explore data distribution and different patterns, which are important in selection of possible models and/or identification of a required transformation. Feasibility was determined by the study (IPTsc) coverage calculated as the number of schoolchildren completing three courses of a DP therapeutic dose divided by the total number of children enrolled at a school/ward in the IPTsc arm. Feasibility was also evaluated by assessment through supervisory reports of teachers’ competence in IPTsc administration with minimal disruption of ongoing school activities. Acceptability was assessed as a qualitative research study, where transcripts and field notes were imported into QSR NVivo 11 qualitative data analysis software for analysis using an inductive and deductive approach.

#### Effectiveness evaluation

Malaria parasite prevalence was calculated as the number of children with any parasites (irrespective of species) on thick smear divided by the total number of children enrolled and tested [37]. Anaemia was defined using the WHO age-specific cut off points for Hb (< 11.5 g/dL for children 6 to < 12 years of age, < 12.0 g/dL for those 12–14 years of age, < 13 g/dL and < 12 g/dL for male and female children aged 15 years, respectively) [37, 38]. Routine clinical evaluation included, malnutrition assessment, where body mass index (BMI) was calculated as (weight(Kg)/height(m)^2^), and the respective anthropometric index z-score, including that of height for age and weight for age, was calculated using the ‘*egen’* STATA function as described elsewhere [37, 39]. Children were classified as underweight/wasted if they were less than two standard deviations (SD) below the reference mean [37].

Malaria prevalence reduction was calculated as the ratio of difference in prevalence achieved in the intervention arm to that of the control arm expressed in percentage. Longitudinal data analysis was performed using a panel data arising from the effectiveness evaluation. Additionally, methods appropriate for a cluster-randomized trial [40, 41] were also used to determine the effect of the intervention stratifying schools into two groups according to baseline malaria prevalence (i.e. above 10% and below). The prevalence, or mean was calculated and the unadjusted risk ratio, or mean difference (intervention–control), estimated in each stratum as described elsewhere [41]. A weighted average of the stratum-specific estimates was used to estimate an overall effect of IPTsc. In addition, measures for comparison of treatment arms were expressed as prevalence, mean difference and protective effect (PE), which was calculated as PE = 1 − (rate ratio of malaria parasitaemia, clinical malaria or anaemia) × 100% as was done elsewhere [41, 42]. Kaplan–Meier analysis was used to estimate the time to the first episode of clinical malaria. The 95% confidence intervals (CIs) were calculated, and a P-value of < 0.05 was considered statistically significant when comparing the IPTsc arm with the control. Data were analysed as intention-to-treat (ITT) in which eligible randomized schoolchildren who participated in any visit were included as per study arms.

## Discussion

This study has outlined an approach that can simplify introduction of IPTsc and possibly other various school health programmes in settings similar to Tanzania. It could also be useful for planning integration of various interventions to reduce the implementation cost, especially on drug delivery and supervision. To implement IPTsc, the study team had to understand how other programmes were implemented. It was noted that successful programme introduction has to consider the targeted population platform (meeting point) and schedule that routinely gather the targeted population and the intended outcome for the planned intervention. For example, the Intermittent Preventive Treatment in pregnancy (IPTp) was introduced under the platform of routine antenatal care (ANC) among women who were receiving antenatal care on monthly basis and has resulted into successful introduction of IPTp-using sulfadoxine pyrimethamine (SP) aiming at prevention of malaria infection during pregnancy and improving birth outcome. The same for IPT in infants (IPTi-SP, now termed as perennial malaria chemoprevention-[PMC]) has been implemented using the routine immunization programme; it also aimed at prevention of infection. A different approach has been used for the seasonal malaria chemoprevention (SMC) that is largely practiced in West Africa in the Sahel region; was designed to prevent infection in children under-five in a period of high season of malaria infection. In this, the gathering platform for children especially after completing the routine infant vaccination schedule was not available; they therefore made use of CHWs who would administer monthly anti-malarial medication (SPAQ) to children in their communities consecutively for 3–4 months. All these aspects have been successfully implemented [43, 44].

When it comes to school-aged children, the study team had to capitalize the fact that nowadays school enrolment in most African countries has significantly improved [45–49] with more than 95% of school-aged children registered in primary schools. Schoolchildren are available in schools throughout the year, and are more likely to be residents of the same area for at least 7 years (the duration of primary school education in Tanzania) before shifting to other areas for secondary school education. Given the recruitment age starts at 5 years it is more likely they graduate by 14–15 years. Thus, from epidemiological point of view and for feasible pragmatic implementation to shrink the malaria reservoir in endemic communities, schools become important platform for delivery of IPTsc. In fact, malaria in schoolchildren highly correlates to malaria infections in pregnant women and under-five children [23, 50], making a plausible hypothesis that IPTsc would likely reduce the burden not only to schoolchildren but also to the communities as was shown elsewhere [51]. In addition, the big advantage of IPTsc compared the others is the service driven approach, (i.e. IPTi and IPTp are dependent on an individual participant to present at a facility, therefore, making coverage lower especially in IPTp [52]), while, IPTsc is organized by the facility staff and supervised by teachers, something you cannot see or arrange for IPTi, or IPTp. Thus, schools may provide a low-cost means to deliver chemoprevention to school-aged children [13].

There are other programmes that run through schools: the school MDA has been widely practiced in African schools [11, 12, 53, 54], they are mostly delivered by school teachers who get trained to administer the drugs and collaborate with local health facilities for management of any adverse event [55]. These teachers can be trained to deliver IPTsc as was done in this study. Pragmatically, the frequency at which IPTsc has to be delivered on annual basis needs to be established. The monthly model (like IPTp) will not be realistic, as it will disrupt school curriculum. Therefore, getting to the timing of high malaria transmission season is essential. This will assure administering the drug at the very point needed to clear the parasites and prevent further infection in the specified malaria season. Thus, areas with two seasonal peaks following rainfall trends would likely need three rounds and those with single seasonal peak per year would require two rounds of IPTsc delivery. Such intervals are likely to be pragmatically feasible, as it will allow teachers to proceed with other curriculum activities.

This study has laid out a proof of concept for operational ability of IPTsc in most endemic communities. The implementation approach and tools used in this study can be customized to fit the purpose in any community/country interested on IPTsc. The conduct of IPTsc was successful despite having some operational challenges that were well mitigated to bring a successful operation worthy adopting by any other nation. The first challenge was on school closure due to COVID-19 pandemic, which led the team to postpone the start of clinical and IPTsc administration visits from March to July–August, 2020, a time that was initially planned to be for round 2 of IPTsc. The shift did not affect the study since no clinical evaluation or drug administration had been conducted, so it was just a push on the plan forward. The monitoring following high rain season that normally starts March–May was then assessed following the third IPTsc round, so there was no strategic interruption on evaluation of IPTsc effectiveness.

The other challenge was in reaching out the communities to explain the study. Since the community is huge and some would not attend briefing meetings, meetings were conducted on each school where parents committee inviting village leaders and key opinion leaders to help propel information in every gathering that they may hold internally. In addition, political leaders such as the District Commissioners were well engaged to the operation of IPTsc, they would deliver a massage on every community gathering they encounter in the event they visited interventional wards. Such involvement of community leaders and political leaders played an important role in the conduct of IPTsc. Issues like school food donation from parents/guardians were as well addressed on every meeting as a message encompassing successful IPTsc administration in their respective schools.

The delivery of a three days anti-malarial regimen is indeed different from a one-day anthelminthic drug. Normally two teachers have been used to administer MDA against STH and schistosomiasis, the two would administer the drugs to all schoolchildren in a school, taking almost the whole day to finish. In this study, the study team invented the wheel by improving the number of teachers to be used per school. Seven teachers (one per class) was set as a default number, but basing on the fact that there are other schools with more schoolchildren enrolled, the number of teachers could reach to 17 per school for those with more than 1400 schoolchildren per school. This was done to enable a teacher to administer IPTsc drugs only for a short duration not disturbing the school curriculum. This made the operation effective since within an hour or two, the whole school would have finished drug administration. Incentive payment was made equally to all teachers involved, and was made once covering all 5 days set for drug dispensing (five covering those who would miss in any day). In addition, two CHWs were included in the list; these were helpful on finding or dosing those who missed school on day of administration. A similar arrangement can be made to access school-aged children who are not enrolled at schools, thus addressing equity concerns.

## Conclusion

IPTsc was feasibly implemented and well accepted by the community, henceforth, through this article other interested countries may realise an applicable approach for IPTsc implementation. Mitigation to any challenge can be customized based on local circumstances without jeopardising the gains expected from an IPTsc programme. After completing the feasibility assessment, the next step was to estimate cost-effectiveness including hours, in-kind investment of teachers, health staff, drugs procurement etc. in a real world (implementation) situation. The study team subsequently, developed an IPTsc scale up plan and a policy brief for the endorsement of IPTsc strategy in Tanzania.

## Supplementary Information

**Additional file 1: Appendix S1. **Drug accountability log.

**Additional file 2: Appendix S2. **Drug dispensing log.

**Additional file 3: Appendix S3. **Drug dispensing information sheet.

**Additional file 4: Appendix S4. **Adverse drug reaction reporting form.

**Additional file 5: Appendix S5. **IPTsc coverage assessment form.

**Additional file 6: Appendix S6. **Supervision form.

**Additional file 7: Appendix S7. **School attendance monitoring form.

**Additional file 8: Appendix S8. **Referral form.

**Additional file 9: Appendix S9. **Clinical episodes recording form.

## Data Availability

All relevant data are within the manuscript and its Supporting Information files. However, in case of further details, data will be available from the corresponding author on reasonable request and approval from the collaborating institutions and signing the data transfer agreement (DTA) from NIMR [36].

## Abbreviations

ACT: Artemisinin-based combination therapy
ADR: Adverse drug reaction
AEs: Adverse events
AL: Artemether-lumefantrine
CRF: Case record form
DBS: Dried blood spot
DP: Dihydroartemisinin-Piperaquine
EMA: European Medicine Agency
GCP: Good Clinical Practice
GHI: Global Health Institute
GMP: Good Manufacturing Practice
Hb: Haemoglobin
IRB: Institutional review board
IRS: Indoor residual spraying
IPTi: Intermittent preventive treatment in infants
IPTp: Intermittent preventive treatment in pregnancy
IPTsc: Intermittent preventive treatment in schoolchildren
ITNs: Insecticide treated nets
ITT: Intention to treat
LLINs: Long-lasting insecticide treated nets
MDA: Mass drug administration
MRCC: Medical Research Coordinating Committee
NSHP: National School Health Programme
NTD: Neglected Tropical Diseases
NIMR: National Institute for Medical Research, Tanzania
NMCP: National Malaria Control Programme
PCR: Polymerase chain reaction
PQ: Piperaquine
Pfkelch13: *Plasmodium falciparum* Kelch-13 gene
*pfcrt*: *Plasmodium falciparum chloroquine resistance transporter*
Pfmdr1: *Plasmodium falciparum* Multidrug-resistance gene-1
RCT: Randomized controlled trial
RDT: Rapid diagnostic test
SAE: Serious adverse event
SOP: Standard operating procedure
SP: Sulfadoxine-Pyrimethamine
STH: Soil transmitted helminths
TMDA: Tanzania Medicines and Medical Devices Authority
DSHCo: District School Health Coordinator
TSHCo: Town School Health Coordinator
DMO: District Medical Officer
TMO: Town council Medical Officer
DNTDCo: District NTD Coordinator
DPEO: District Primary Education Officer
TPEO: Town council Primary Education Officer. DPharm: District Pharmacist
TPharm: Town council Pharmacist
