# Supplementary material for: Implementation research of a cluster randomized trial evaluating the implementation and effectiveness of intermittent preventive treatment for malaria using dihydroartemisinin-piperaquine on reducing malaria burden in school-aged children in Tanzania: methodology, challenges, and mitigation
Source: Malar J. 2023 Jan 6;22:7. doi: 10.1186/s12936-022-04428-8 (PMC9816525; doi:10.1186/s12936-022-04428-8)
Supplement: Supplementary file 1 — Additional file 1: Appendix S1. Drug accountability log. [file 12936_2022_4428_MOESM1_ESM.pdf]

# IPTsc study. Handeni (DC&TC) and Kilindi DC, TANGA.

**DRUG ACCOUNTABILITY LOG**

DRUGS RECEIPT AND DISPENSING

| | |
|---|---|
| PROTOCOL ID | IPTsc version 1 dated 19th Jul 2019 |
| SITE | ( ) Handeni TC ( ) Handeni DC ( ) Kilindi DC<br>Name of health facility / school: ________________Ward:___________________Facility in charge officer:_______________________ |
| **DRUG details** | **please check one (please use one form per batch number)**<br>**( ) DP Trade name: Strength: Batch number: Expirely date:**<br>**( ) Other Trade name: Strength: Batch number: Expirely date:** |

IPTsc Drug description of a blister pack: ( ) 6 tablets ( ) 9 tablets (*put a tick*)

| **Received** | | | | **Dispensed to field / other facilities /school / class** | | | | | | **Returned** | | |
|---|---|---|---|---|---|---|---|---|---|---|---|---|
| Number of Boxes /doses received | Supplier name | Received by (initials) | Date (dd-mmm-yyyy) | Dispensed to (facility name) | Number of boxes /doses dispensed | Date (dd-mmm-yyyy) | Received by (initials) | Product condition | Balance | Number of tablets | Date (dd-mmm-yyyy) | Counted by (initials) |
| | | | | | | | | | | | | |
| | | | | | | | | | | | | |
| | | | | | | | | | | | | |
| | | | | | | | | | | | | |
| | | | | | | | | | | | | |
| | | | | | | | | | | | | |
| | | | | | | | | | | | | |
| | | | | | | | | | | | | |
| | | | | | | | | | | | | |
| | | | | | | | | | | | | |
| | | | | | | | | | | | | |
| | | | | | | | | | | | | |
| | | | | | | | | | | | | |
| | | | | | | | | | | | | |
