# Supplementary material for: Implementation research of a cluster randomized trial evaluating the implementation and effectiveness of intermittent preventive treatment for malaria using dihydroartemisinin-piperaquine on reducing malaria burden in school-aged children in Tanzania: methodology, challenges, and mitigation
Source: Malar J. 2023 Jan 6;22:7. doi: 10.1186/s12936-022-04428-8 (PMC9816525; doi:10.1186/s12936-022-04428-8)
Supplement: Supplementary file 2 — Additional file 2: Appendix S2. Drug dispensing log. [file 12936_2022_4428_MOESM2_ESM.pdf]

IPTsc DRUG DISPENSING AND ACCOUNTABILITY LOG

Form number 1: Version 1 _ 02-July-2020.

**PROTOCOL ID**	**IPTsc version 1 dated 19 Jul 2019**		
**SITE**	**( ) Handeni TC**	**( ) Handeni DC**	**( ) Kilindi DC** ***(put a tick)***
	**School name:**	**Ward name:**	**Teacher's name:**
**CLASS INFORMATION**	**Class:**	**Total number of pupils in this class:**	
**THE DRUG**	***please use one form for each batch number***		
	**( ) DP Trade name:**	**Strength:**	**Batch number:**

Student name (as per school attendance register)		Sex (M/F)	Weight (Kg)	Age (yrs)	No of tablets administered	Date of dose day 1	Dose day (tick)			Date of dose day 3	AE reported (Y/N)*	Missing**
							1	2	3			

Total number of tablets ____________ *Total number of pupils completed dose:*______________________

*Total number of pupils with incompleted dose:*________________

** If yes fill the "yellow form" from TMDA*

***HAK=Hakupata dawa (Did not receive drug), 1=Amekataa (Refused), 2=Mgonjwa (Sick), 3=Hakuhudhuria (Absent), 4=Amehama shule (Shifted school), 5=Amefariki (Died), 6= Ana umri chini ya miaka 5 (Age below 5 years), 7=Vomited more than twice*

Page number_____of______
