# Supplementary material for: Implementation research of a cluster randomized trial evaluating the implementation and effectiveness of intermittent preventive treatment for malaria using dihydroartemisinin-piperaquine on reducing malaria burden in school-aged children in Tanzania: methodology, challenges, and mitigation
Source: Malar J. 2023 Jan 6;22:7. doi: 10.1186/s12936-022-04428-8 (PMC9816525; doi:10.1186/s12936-022-04428-8)
Supplement: Supplementary file 3 — Additional file 3: Appendix S3. Drug dispensing information sheet. [file 12936_2022_4428_MOESM3_ESM.pdf]

DIHYDROARTEMISININ/PIPERAQUINE = DP (or DHAPPQ) oral

– For this study, the drug is available in blister packs as follows:

- 40 mg DHA/320 mg PPQ tablets   blister pack of 6 tablets
- 40 mg DHA/320 mg PPQ tablets   blister pack of 9 tablets

Dosage and duration

– The study drugs are administered basing on participant's weight following manufacturer's instruction (package insert/leaflet). A three days course treatment, once daily is narrated on the table below.

Weight	**Day1** (40 mg/320 mg tablet)	**Day2** (40 mg/320 mg tablet)	**Day3** (40 mg/320 mg tablet)
11 to < 17 kg	1 tab	1 tab	1 tab
17 to < 25 kg	1½ tab	1½ tab	1½ tab
25 to < 36 kg	2 tab	2 tab	2 tab
36 to < 60 kg	3 tab	3 tab	3 tab
60 to < 80 kg	4 tab	4 tab	4 tab
≥ 80 kg	5 tab	5 tab	5 tab

Do not give a child drugs if: (Contraindication to medication)
–A child has a known cardiovascular condition or is currently using medication for cardiovascular disease.
–If a child is on antimalarial medication or has completed antimalarial treatment within a week preceding IPTsc dispensing.
--If a child is known to be allergic to ACT drugs (Dawa za mseto) for malaria.

Drug dispensing procedures

- ✓ Before dispensing drugs, Batch number and expiry date should be keenly observed and fill accordingly on the dispensing forms.
- ✓ Dispense following the weight of a child
- ✓ Drugs should be taken with water before meals (preferably in between meals). In case drug dispensing goes beyond lunch hour, children can still take their medication post lunch.
- ✓ Children should be observed for 30 minutes after ingesting the study drug. If a child vomits within this time period, let him/ her rest for a while and give another course (e.g. 1 tab as was supposed to take) only to repeat once and the information documented on dispensing form. If one vomits beyond half an hour but within one

hour, the child should be given half a dose (e.g. ½ tab an amount initially supposed to take for the day). However, if a child vomits the study drug for the second time, he or she is withdrawn from receiving the study drug.

- ✓ Patience and creativity should be taken to encourage the younger children take the medication.

- ✓ If a child misses day-one dose, he/ she should start on the second day. In the event a child misses day 2 dose, one should be fetched to take the dose as scheduled and could take it on the third day and so finish the third course on the fourth day. However, if a child misses dosing for two consecutive days, then one will be regarded as incomplete dose and documented as such.

- ✓ Drugs should be given to all children aged 5 years and above as long as a child is in that school. Children not registered in the respective primary school will not receive the drug under this school.

- ✓ Drugs should be stored in a cool dry place, preferably below *25 °C* - - , kept away from direct sunlight, rain and humidity.

In case an adverse drug reaction (ADR) occur

- ✓ Inform the clinical officer at the centre where you took drugs for this school and fill in the TMDA yellow form

- ✓ All medical decisions including if stopping of medication is required, should come from the clinical officer in a nearby hospital (preferably the centre where drugs were taken). The health facility will also manage all medical condition (ADRs), at a cost of the IPTsc programme.

Contact for further clarification

- ✓ In case of any further clarification, ask the clinician at the health facility where you took the drugs, contact are provided below.

- ✓ ***Ward name***:……………………..

- ✓ Name of health facility in charge:……………………….

- ✓ Mobile phone number of a clinician:…………………….
