# Supplementary material for: Implementation research of a cluster randomized trial evaluating the implementation and effectiveness of intermittent preventive treatment for malaria using dihydroartemisinin-piperaquine on reducing malaria burden in school-aged children in Tanzania: methodology, challenges, and mitigation
Source: Malar J. 2023 Jan 6;22:7. doi: 10.1186/s12936-022-04428-8 (PMC9816525; doi:10.1186/s12936-022-04428-8)
Supplement: Supplementary file 4 — Additional file 4: Appendix S4. Adverse drug reaction reporting form. [file 12936_2022_4428_MOESM4_ESM.pdf]

THE UNITED REPUBLIC OF TANZANIA
MINISTRY OF HEALTH, COMMUNITY DEVELOPMENT, GENDER, ELDERLY AND CHILDREN

TMDA
Tanzania Medicines & Medical Devices Authority

TANZANIA MEDICINES AND MEDICAL DEVICES AUTHORITY

ADR REPORTING FORM

(Made under regulations 33, 36(1)(a), 38(1), and 46(1)

Note: Reporters and patients identity are held in strict confidence by TMDA and protected to the fullest extent of the law

Type of Report

Initial ❑ Follow up ❑ Serious ❑ Not Serious❑ Medical ❑ Vaccine ❑

Patient Information

Patient ID/ Initials.: - ___________________ Gender: Male ❑ Female ❑ Weight(kg)………..Pregnancy status Yes ❑ No ❑

Full address …………………………. Telephone Number …………………………….

Date of Birth : ../…/…(dd-mm-yyyy) OR Age at onset: ___________

Medical History (Provide any relevant medical history and laboratory results including dates (if done)

……

………..

Details of suspected medical product

**Name of suspected medicine (s) (Specify brand name or manufacturer if known)**	**Generic name**	**Route**	**Dose and frequency**	**Therapy Date**		**Batch. No & Expiry date (If known)**	**Indication (Reason for use)**
				**Date stated**	**Date Stopped**		
1.							
2.							
**Other medicines used at the same time and or one month before (including herbal medicines)**							
1.							
2.							

Date of ADR onset:../…../…… Time of onset ……..Date ADR stopped………/…./………

Severity **of** the ADR

Mild ❑ Moderate ❑ Severe ❑ Fatal ❑ Unknown ❑

Reasons for seriousness

Prolonged hospitalization ❑ Caused a congenital anomaly ❑ Disability ❑ Death ❑ Life threatening ❑

Action taken

Dose increased ❑ Dose reduced ❑ Dose changed ❑ Not applicable ❑ Unknown ❑

Outcome

Recovering ❑ Recovered with sequalae ❑ Not recovered ❑ Death ❑ Unknown ❑ Recovered ❑

Causality of the ADR/AEFI

Certain ❑ Probable/Likely ❑ Possible ❑ Unlikely ❑ Unclassifiable ❑

Therapeutic failure (provide information on medicine(s)/vaccine(s) showed lack of efficacy

Medication errors (provide detail of medication errors)

:

Additional Information (Provide any other relevant additional information below)

Administrative information

Report Title	Form ID number:	Date of reporting
Name and Address of Institution	Email Address	Country
Name of reporter :	Contact /Tel No:	Email

Thank you for your cooperation

GUIDANCE ON REPORTING

What to report

Report all adverse drug reactions/events suspected both serous and those that are not serious.

Report any adverse reaction or AEFIs even if you are not certain the prod- uct caused the event

When To Report

For serious ADRs within 24-48 hrs.' of notification For

AEFIs report immediately you are notified

For non-serious events as soon as possible but not later than 15 days

Who Is To Report

- All Healthcare Providers should report as part of their professional responsibility any suspected adverse drug reactions and AEFIs
- Where To Report
- reports should be sent to the NMRAs
- reports can also be sent to the national AEFI committee

How to report

- fill in the sections that apply to your report
- Start date of administration for the suspected drug and the date when the suspected reaction occurred

Severity of reaction

Mild: ADR/AEFI that requires no change intreatment with the suspected drug. Requires suspected drug to be withheld, discontinued or otherwise changed. No prolonged hospitalization

Moderate: ADR/AEFI requires the suspected drug to be withheld, dis- continued or otherwise changed. Prolongs hospitalization by at least 1 day. ADR is the reason for admission

Severe: ADR/AEFI requires intensive medical care, causes permanent harm to the patient

Fatal: ADR/AEFI either directly or indirectly leads to death of the

patient **Detection of ADR/AEFIs in a Patient**

Follo

w the following steps

Take proper history and conduct proper examination of the patient.

Ensure that the medicine ordered is the medicine received and actually taken by the patient at the dose advised.

Verify that the onset of the suspected ADR was after the drug was taken, not before and discuss carefully the observation made by the patient.

Determine the time interval between the beginning of drug treatment and the onset of the event.

- Evaluate the suspected ADR after discontinuing the drugs or reducing the dose and monitor the patient's status (De- challenge). If appropriate, restart the drug treatment and monitor recurrence of any adverse events (Re-challenge).
- Analyze the alternative causes (other than the drug) that could on their own have caused the reaction.
- Use relevant up-to date literature and personal experience as a health professional on drugs and their ADRs and verify if there are previous conclusive reports on this reaction

Please note that submission of a report doesn't imply that the health worker or the product caused or contributed to the adverse event

WHO-UMC causality assessment scale

Causalit y Term	Assessment
Certain	• Event of laboratory test abnormality, with plausible time relationship to drug intake<br>• Cannot be explained by disease or other drugs<br>• Response to withdrawal plausible (pharmacologically, pathologically)<br>• Event definitive pharmacologically or phenomenologically *(i.e. an objective and specific medical disorder or a recognized pharmacological phe- nomenon)*<br>• Rechallenges at is factory, if necessary
Probable	• Event or laboratory test abnormality, with reasonable time relationship to drug intake.<br>• Unlikely to be attributed to disease or other drugs<br>• Response to withdrawal clinically reasonable<br>• Rechallenge not required
Possible / likely	• Event or laboratory test abnormality, with reasonable time relationship to drug intake<br>• Could also be explained by disease or other drugs<br>• Information on drugs withdrawal lacking or unclear
Unlikely	• Event or laboratory tests abnormality, with a time to drug intake that makes a relationship improbable (but not impossible)<br>• Disease or other drugs provide plausible explanations
Conditional / Unclassified	• Event or laboratory test abnormality<br>• More data for proper, assessment needed or<br>• Additional data under examination
Un assessable / unclassifiable	• Report suggesting an adverse reaction<br>• Cannot be judged because of insufficient or contradictory information<br>• Data cannot be supplemented or verified

Confidentiality

All information pertaining to the reported event should at all times be treated in confidence and protected from an authorized access transmission of use.
