# Supplementary material for: Implementation research of a cluster randomized trial evaluating the implementation and effectiveness of intermittent preventive treatment for malaria using dihydroartemisinin-piperaquine on reducing malaria burden in school-aged children in Tanzania: methodology, challenges, and mitigation
Source: Malar J. 2023 Jan 6;22:7. doi: 10.1186/s12936-022-04428-8 (PMC9816525; doi:10.1186/s12936-022-04428-8)
Supplement: Supplementary file 5 — Additional file 5: Appendix S5. IPTsc coverage assessment form. [file 12936_2022_4428_MOESM5_ESM.pdf]

IPTsc DRUG DISPENSING : ACCOUNTABILITY AND COVERAGE ASSESSMENT

Form number 2: Version 1 _ 05-July-2020.

**PROTOCOL ID**	**IPTsc version 1 dated 19 Jul 2019**		
**District**	**( ) Handeni TC ( ) Handeni DC ( ) Kilindi DC** ***(put a tick)***		
**Person filling this form**	**Name:** **Position:**		
	Information on IPTsc coverage in a class or school or ward. Should be filled with class teacher / head teacher/ or ward education officer of a respective ward.	***Sex*** ***Ma***	***Fe***
**Study area**	**Name of a Class / School /Ward:** **Total number of pupils in a class / school / ward:**		
**IPTsc Implementation information**	**A. Total number of schoolchildren who completed DP dose** ________________	________	________
	**B. Total number of schoolchildren who did not complete DP dose** ________________	________	________
	**C. Total number of tablets used** ________________	________	________
	**D. Total nmber of schoolchildren who missed dose for diffrerent reasons** ________________	________	________
	Reasons **1) Refused** ________________	________	________
	**2) Sick** ________________	________	________
	**3) Absent** ________________	________	________
	**4)Transfer out** _________________	________	________
	**5) Died** _________________	________	________
	**6) Age below 5 years old** _________________	________	________
	**7) Vomited 2 times** __________________	________	________
