# Supplementary material for: Implementation research of a cluster randomized trial evaluating the implementation and effectiveness of intermittent preventive treatment for malaria using dihydroartemisinin-piperaquine on reducing malaria burden in school-aged children in Tanzania: methodology, challenges, and mitigation
Source: Malar J. 2023 Jan 6;22:7. doi: 10.1186/s12936-022-04428-8 (PMC9816525; doi:10.1186/s12936-022-04428-8)
Supplement: Supplementary file 6 — Additional file 6: Appendix S6. Supervision form. [file 12936_2022_4428_MOESM6_ESM.pdf]

Intermittent Preventive Treatment of malaria in schoolchildren (IPTsc)
Handeni DC & TC na Kilindi DC

District:……………………… **Date of supervision:…………………………..** **IPTsc round (   ) 1, (   ) 2, (   ) 3**

Name of the supervisor:______________________________

Summary of Qualitative IPTsc Supervision			***IPTsc with DP***	
**Ward**	**School name**	**Filling in of drug dispensing forms**<br>*(#1,2 and yellow form)*<br>*-[Outline any flaws or inconsistence observed]*	**DP drug administration**<br>*-[outline any flaws or inconsistences e.g. dose and weight]*	**Suggestions or any challenge noted**

Signature of the supervisor:…………………………………… **Name and signature of a respective WEO:……………………………………………………………….**
