# Supplementary figures and images for: Implementation research of a cluster randomized trial evaluating the implementation and effectiveness of intermittent preventive treatment for malaria using dihydroartemisinin-piperaquine on reducing malaria burden in school-aged children in Tanzania: methodology, challenges, and mitigation
Source: Malar J. 2023 Jan 6;22:7. doi: 10.1186/s12936-022-04428-8 (PMC9816525; doi:10.1186/s12936-022-04428-8)

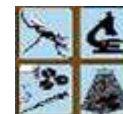

(IPTsc-School project)

| MONTH/YEAR<br>(mmm/yyyy) | SCHOOL NAME | CLASS |
|--------------------------|-------------|-------|
|--------------------------|-------------|-------|

[illegible]

Page number \_\_\_\_ of \_\_\_\_

Supplement: Supplementary file 7 — Additional file 7: Appendix S7. School attendance monitoring form. [file 12936_2022_4428_MOESM7_ESM.pdf]
