# Supplementary material for: Implementation research of a cluster randomized trial evaluating the implementation and effectiveness of intermittent preventive treatment for malaria using dihydroartemisinin-piperaquine on reducing malaria burden in school-aged children in Tanzania: methodology, challenges, and mitigation
Source: Malar J. 2023 Jan 6;22:7. doi: 10.1186/s12936-022-04428-8 (PMC9816525; doi:10.1186/s12936-022-04428-8)
Supplement: Supplementary file 8 — Additional file 8: Appendix S8. Referral form. [file 12936_2022_4428_MOESM8_ESM.pdf]

IPTsc STUDY

Evaluating the implementation and effectiveness of intermittent preventive treatment for malaria in schoolchildren

Referral form

Name of patient: ____

Sex: Fe______/Ma_______ Age (years):_________ Weight:(Kg)_______

Ward:__________________School:___________________Hamlet:______________________

Medical history: ____

____

____

mRDT results: Positive (+VE)_____________/ Negative (-VE)__________________________

Treatment given:____

____

Reason for refferal:____

____

Name of community health care worker:____________________________

Signature:___________________________________

Date:__________________________________
