# Supplementary material for: Implementation research of a cluster randomized trial evaluating the implementation and effectiveness of intermittent preventive treatment for malaria using dihydroartemisinin-piperaquine on reducing malaria burden in school-aged children in Tanzania: methodology, challenges, and mitigation
Source: Malar J. 2023 Jan 6;22:7. doi: 10.1186/s12936-022-04428-8 (PMC9816525; doi:10.1186/s12936-022-04428-8)
Supplement: Supplementary file 9 — Additional file 9: Appendix S9. Clinical episodes recording form. [file 12936_2022_4428_MOESM9_ESM.pdf]

Name of CHW:______________________________ Ward:_______________________School:________________Health facility:__________________

NATIONAL INSTITUTE FOR MEDICAL RESEARCH (NIMR)												
**Intermittent preventive treatment for malaria in school children (IPTsc) programme.**												
**Malaria episode record follow up form**												
**Sn**	**Date**	**Name of child**	**ID No**	**Sex**	**Age**	**School**	**Hamlet**	**mRDT results**	**Diagnosis**	**Treatment**	**Parent's or guardian's signature**	**Parent's or guardian's phone #**

Name and signature of a supervising clinician /Name of health facility:_____________________________________
